# Divergent age-dependent peripheral immune transcriptomic profile following traumatic brain injury

**DOI:** 10.1038/s41598-019-45089-z

**Published:** 2019-06-12

**Authors:** Amanda Hazy, Lauren Bochicchio, Andrea Oliver, Eric Xie, Shuo Geng, Thomas Brickler, Hehuang Xie, Liwu Li, Irving C. Allen, Michelle H. Theus

**Affiliations:** 1 The Department of Biomedical Sciences and Pathobiology, Virginia Tech, 970 Washington Street, Blacksburg, VA 24061 USA; 2 The Department of Statistics, Virginia Tech, 250 Drillfield Drive, Blacksburg, VA 24061 USA; 3 Biocomplexity Institute, Virginia Tech, 1015 Life Science Circle, Blacksburg, VA 24061 USA; 4 School of Neuroscience, Virginia Tech, Blacksburg, VA 24061 USA; 5 Center for Regenerative Medicine, College of Veterinary Medicine, Blacksburg, Virginia 24061 USA; 6 The Department of Biological Sciences, College of Science, Virginia Tech, 970 Washington Street, Blacksburg, VA 24061 USA

**Keywords:** Neuroimmunology, Molecular neuroscience

## Abstract

The peripheral immune system is a major regulator of the pathophysiology associated with traumatic brain injury (TBI). While age-at-injury influences recovery from TBI, the differential effects on the peripheral immune response remain unknown. Here, we investigated the effects of TBI on gene expression changes in murine whole blood using RNAseq analysis, gene ontology and network topology-based key driver analysis. Genome-wide comparison of CCI-injured peripheral whole blood showed a significant increase in genes involved in proteolysis and oxidative-reduction processes in juvenile compared to adult. Conversely, a greater number of genes, involved in migration, cytokine-mediated signaling and adhesion, were found reduced in CCI-injured juvenile compared to CCI-injured adult immune cells. Key driver analysis also identified G-protein coupled and novel pattern recognition receptor (PRR), P2RY10, as a central regulator of these genes. Lastly, we found Dectin-1, a c-type lectin PRR to be reduced at the protein level in both naïve neutrophils and on infiltrating immune cells in the CCI-injured juvenile cortex. These findings demonstrate a distinct peripheral inflammatory profile in juvenile mice, which may impact the injury and repair response to brain trauma.

## Introduction

Traumatic brain injury (TBI) is a leading cause of death and permanent neurological disability across the age spectrum. It is well-established that peripheral immune mechanisms are involved in the acute and chronic pathophysiological outcome of TBI and this response may be age-dependent^1–3^. Inflammation originating in the brain has a profound effect on the kinetics of leukocyte recruitment, which has been shown to be differentially regulated as the developing brain is more amenable to inflammatory stimuli than the adult brain^4,5^. Following blood brain barrier (BBB) disruption, early activation and infiltration of neutrophils, dendritic cells and macrophages contributes to the neurotoxic milieu by providing both pro- and anti-inflammatory responses. Inflammation is a major driver of tissue damage and repair in acute and chronic TBI. It is part of the complex pathogenesis that includes reactive oxygen species, disruption of ion and calcium homeostasis, purinergic receptor signaling, excitotoxicity, BBB disruption, hypoxia, damage-associated molecular pattern molecules and others^6,7^. The early neuroinflammatory response to TBI occurs primarily through innate immune mechanisms. Damage to the brain triggers the release and production of cytokines and chemokines that result in local and systemic inflammation aimed at limiting the spread of injury and restoring homeostatic balance.

While our understanding of the inflammatory response to TBI remains under intense investigation, immune cell recruitment is known to be a necessary process in restoring tissue homeostasis by sequestration of tissue-damaging irritants and dead cell debris from necrotic spillover of intracellular components. However, overzealous inflammation provoked by DAMPS and alarmins, etc mediate production of neurotoxic cytokines and chemokines which exacerbate secondary injury including free radical and oxidative damage^8–10^. Elevated cytokine production is one of the strongest prognostic indicators of poor clinical outcomes in TBI and emerging evidence suggest that targeting aspects of the immune response may offer promising treatment strategies for TBI^11,12^. Moreover, there is evidence that this response may be age-dependent, however, it remains unclear how this may mitigate or impede the evolution of injury.

It is well-established that the developing brain exhibits greater vulnerability to physical trauma as key maturation events may be interrupted resulting in chronic, long-lasting cognitive and/or motor deficits^13,14^. However, recently we observed a paradoxical early neuroprotection following cortical impact in the juvenile murine brain compared to adults that is mediated, in part, by vascular stability following focal cortical impact^15^. It remains unclear whether the immature immune system may display a distinct response to brain injury which could be temporally regulated. Here we consider how age affects the status of the peripheral immune transcriptome under sham and TBI conditions. These findings contribute to our understanding of how the maturation stage of the immune system could affect TBI outcome and whether the divergent pathways elicited in the response to TBI are central to the induction and resolution of injury.

## Results

### Age-dependent comparative transcriptomic analysis of the peripheral whole blood response following sham and controlled cortical impact (CCI) injury

**Figure 1:**
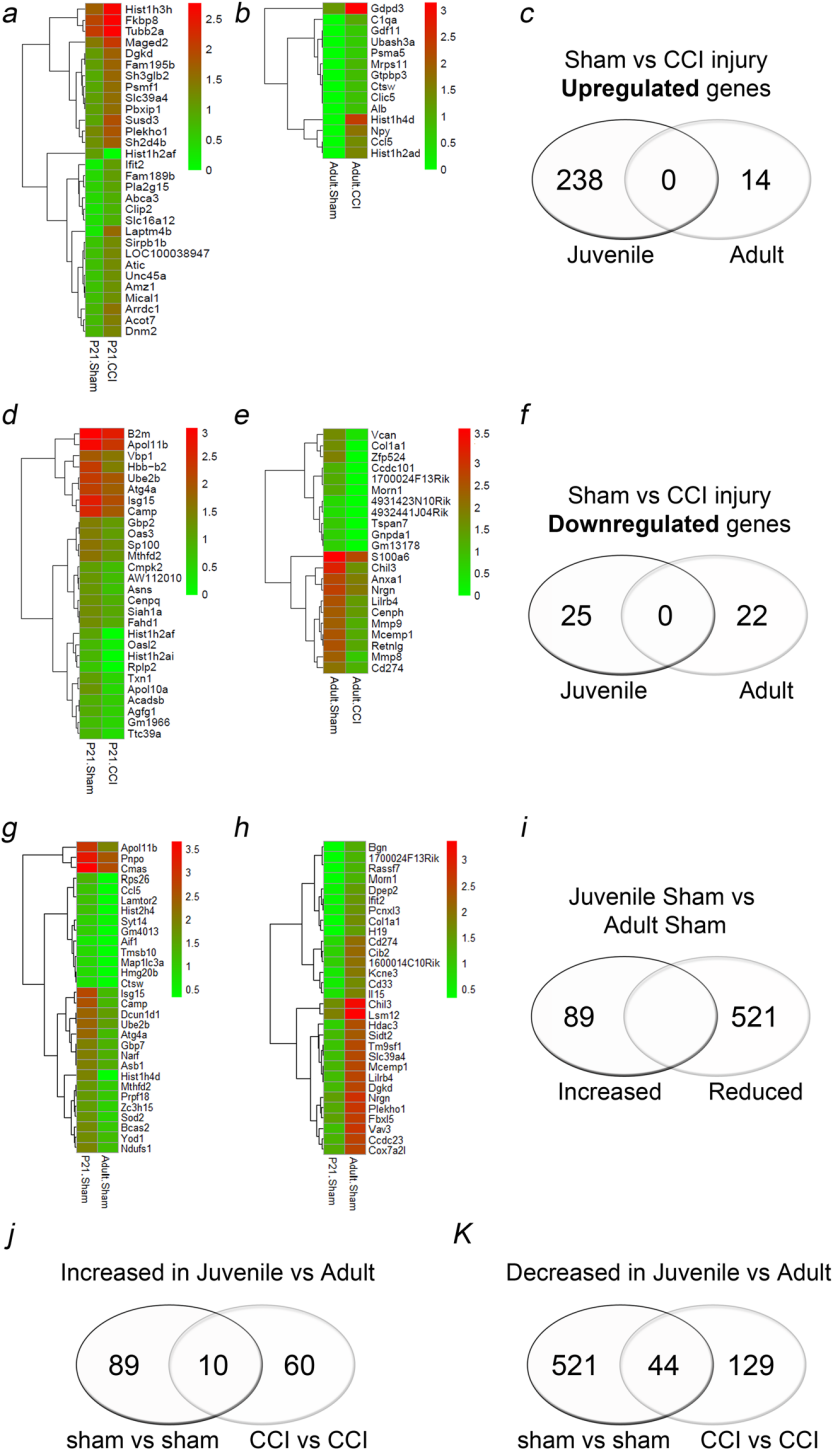
Age-dependent comparative transcriptomic analysis of the peripheral whole blood. (**a**) Heat map of top 30 genes significantly upregulated in the juvenile whole blood at 4d post-CCI injury compared to juvenile sham. (**b**) Identification of Genes upregulated in the adult CCI-injured whole blood compared to sham injury. (**c**) Comparison of genes upregulated following CCI injury in juvenile vs adult whole blood. No upregulated genes were found to overlap. (**d**) Downregulated genes in juvenile CCI-injured and in (**e**) adult whole blood compared to respective shams. (**f**) No downregulated genes were found to overlap. (**g**–**i**) Comparison of genes found to be significantly increased or reduced in sham juvenile compared to sham adult. (**j**) Genes that were increased in juvenile sham vs adult sham that were also increased in juvenile CCI vs adult CCI samples. (**k**) Genes that were decreased in juvenile sham vs adult sham that were also increased in juvenile CCI vs adult CCI samples.

Recent evidence suggests the inflammatory response may contribute to TBI vulnerability and the magnitude of this response may be age dependent^3^. We addressed these potential differences using blood cell fraction transcriptomic analysis in the sub-acute phase of injury or 4 days post-CCI which may reveal changes in acute cell death and/or injury resolution. We observed the juvenile peripheral immune cells display a more dynamic response to CCI injury at the level of gene expression compared to adult cells. Comparative transcriptomic analysis by RNA-sequencing found 238 genes with increased expression and 25 genes with decreased expression in juvenile CCI-injured blood vs sham, compared to only 14 genes with increased expression and 22 genes with decreased expression in adult CCI-injured whole blood vs sham (Fig. 1a–f). The specific transcriptomic response to CCI injury in juvenile mice was distinct from the response observed in adult mice, as no genes found to be increased or decreased following CCI injury overlapped between them. Changes in expression were represented as a heat map. Genes that showed the greatest (>3 log^2^fold) increase in the juvenile whole blood included *Laptm4b* (5.66 log^2^fold), *Ifit2* (4.28 log^2^fold), *Rgs14* (3.27 log^2^fold), *Hist1h3h* (3.18 log^2^fold), *Susd3* (3.18 log^2^fold) and *Phc2* (3.067 log^2^fold). On the other hand, genes with >3 log^2^fold reduction in expression were *Oasl2* (−3.41 log^2^fold) and *Apol10a* (−3.08 log^2^fold). Interestingly, the largest differences were found between uninjured juvenile and adult whole blood at 4 day post-sham surgery. There were 521 genes significantly reduced and 89 genes were increased in juvenile vs adult sham whole blood (Fig. 1i).

Finally, we evaluated which genes were either increased or decreased in the juvenile whole blood between adult sham comparisons vs adult CCI comparisons and whether there was common overlap. We identified 10 genes that were increased in the juvenile in both sham and CCI comparison, including *Sod2, Ltf, Atg4a* (Fig. 1j). We also identified 44 genes that were increased in the juvenile in both sham and CCI comparison, including *Hdac3, Gad1, Cbx4* (Fig. 1h). A complete list of these genes is provided in Supplementary Table 1.

### Age-dependent gene ontology analysis of CCI-injured peripheral immune system

**Figure 2:**
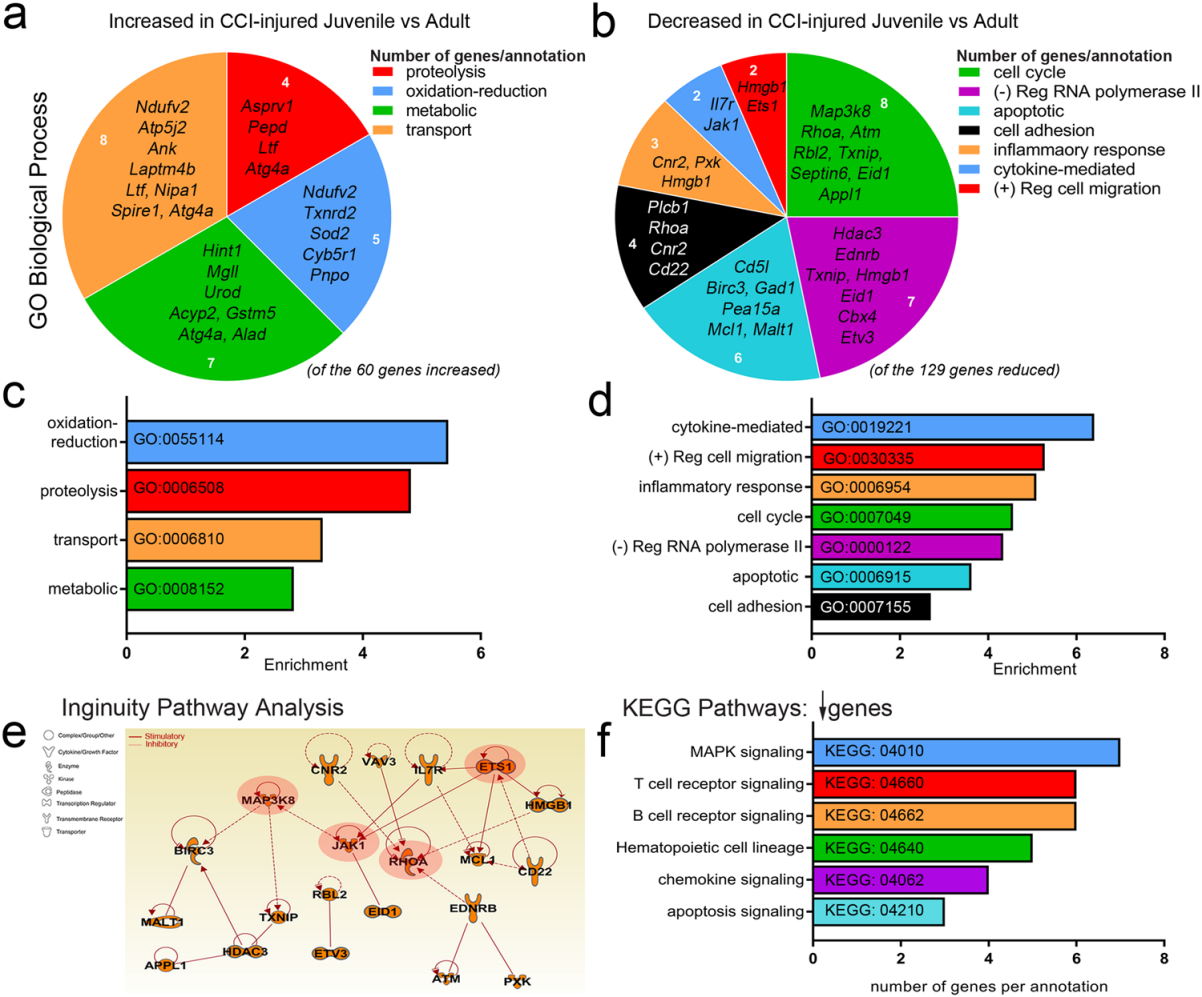
Age-dependent Gene Ontology analysis of CCI-injured peripheral immune response. (**a**,**b**) GO biological process groupings of changing genes show differentially regulated pathways in juvenile vs adult CCI-injured mice. Increased genes were identified in the categories of transport (p = 0.0002), metabolic process (p = 0.0033), oxidation-reduction (p = 1.85E-05) and proteolysis (p = 0.0005). Decreased genes were identified in the categories of cell cycle (p = 8.18E-07), RNA polymerase (p = 9.02E-06), apoptotic processes (p = 0.0004), cell adhesion (p = 0.0274), inflammatory response (p = 0.0011), cytokine-mediated signaling (p = 0.0018) and cell migration (p = 0.0063). (**c**,**d**) Enrichment analysis of GO biological process categories for significantly changing genes in juvenile vs adult CCI-injured mice. (**e**) Ingenuity Pathway Analysis (IPA) shows that MAP3K8, JAK1, ETS1 and RHO (red highlight), among others, represent predicted signaling pathways identified as central to the functions of the down-regulated genes confirmed from above ontology analysis. Interaction of downregulated genes in CCI-injured juvenile vs adult. Networks were generated through the use of IPA: https://www.qiagenbioinformatics.com/products/ingenuity-pathway-analysis ^42^. (**f**) Enrichment analysis of KEGG categories for significantly changing genes in juvenile vs adult CCI-injured mice. P values were calculated using the chi-square method.

Next, we evaluated the mRNA levels between CCI-injured juvenile and adult whole blood. We identified 60 genes that were increased and 129 genes that were reduced in the juvenile compared to adult at 4d post-CCI injury. Gene ontology analysis of these changes, using GeneCodis^16–18^, showed differentially regulated GO biological processes in juvenile compared to adult. Of the genes increased in juvenile CCI-injured whole blood, we found them to associate with oxidation-reduction (5 genes, ex. *Sod2*), proteolysis (4 genes, ex. *Atg4a)*, transport (8 genes, ex. *Laptm4b)* and metabolic processes (7 genes, ex. *Urod)* (Fig. 2a,c). Interestingly, lysosomal-associated protein transmembrane, LAPTM4B a potential oncogene shown to enhance AKT activation^19^ is the only one of 238 genes increased in the juvenile CCI-injured whole blood compared to juvenile sham that is also elevated in the juvenile CCI-injured over adult CCI-injured samples (Fig. 1a). Conversely, we found the reduced genes to be associated with the following GO biological processes: cytokine-mediated signaling (2 genes, ex. *Il7r* and *Jak1*), positive regulation of cell migration (2 genes, ex. *Ets1*), inflammatory response (3 genes, ex. *Cnr2*), cell cycle (8 genes, ex. *Map3k8*), negative regulation of RNA polymerase II (7 genes, ex. *Hdac3*), apoptosis (6 genes, ex. apoptosis inhibitor of macrophage (AIM)/*Cd5L*) and cell adhesion (4 genes, ex. *Rhoa*) (Fig. 2b,d). Ingenuity Pathway Analysis (IPA) of pathways and interactions associated with the genes identified revealed that none of the up-regulated genes were associated beyond the biological processes identified (data not shown). However, IPA revealed that the majority of genes down-regulated in the juvenile following injury either directly or indirectly interact with each other (Fig. 2e). Indeed, 4 downregulated genes were identified as critical signaling molecules with pathway interactions in the juveniles: *MAP3K8*; *JAK1*; *RHOA*; and *ETS1* (Fig. 2e). Lastly, KEGG pathways analysis revealed MAPK, T and B cell receptor, chemokine and apoptosis signaling to be highly enriched molecular pathways amongst the 129 reduced genes. Of these, none overlapped with the 25 downregulated genes found between juvenile sham compared to juvenile CCI-injured samples. These data suggest the juvenile CCI-injured peripheral immune system may display an enhanced response against oxidative stress and reduced inflammatory, migratory and apoptotic processes. Furthermore, these signaling differences may be related to T and B-cell development and function.

### P2ry10 is a key driver of the juvenile gene profile following CCI injury

**Figure 3:**
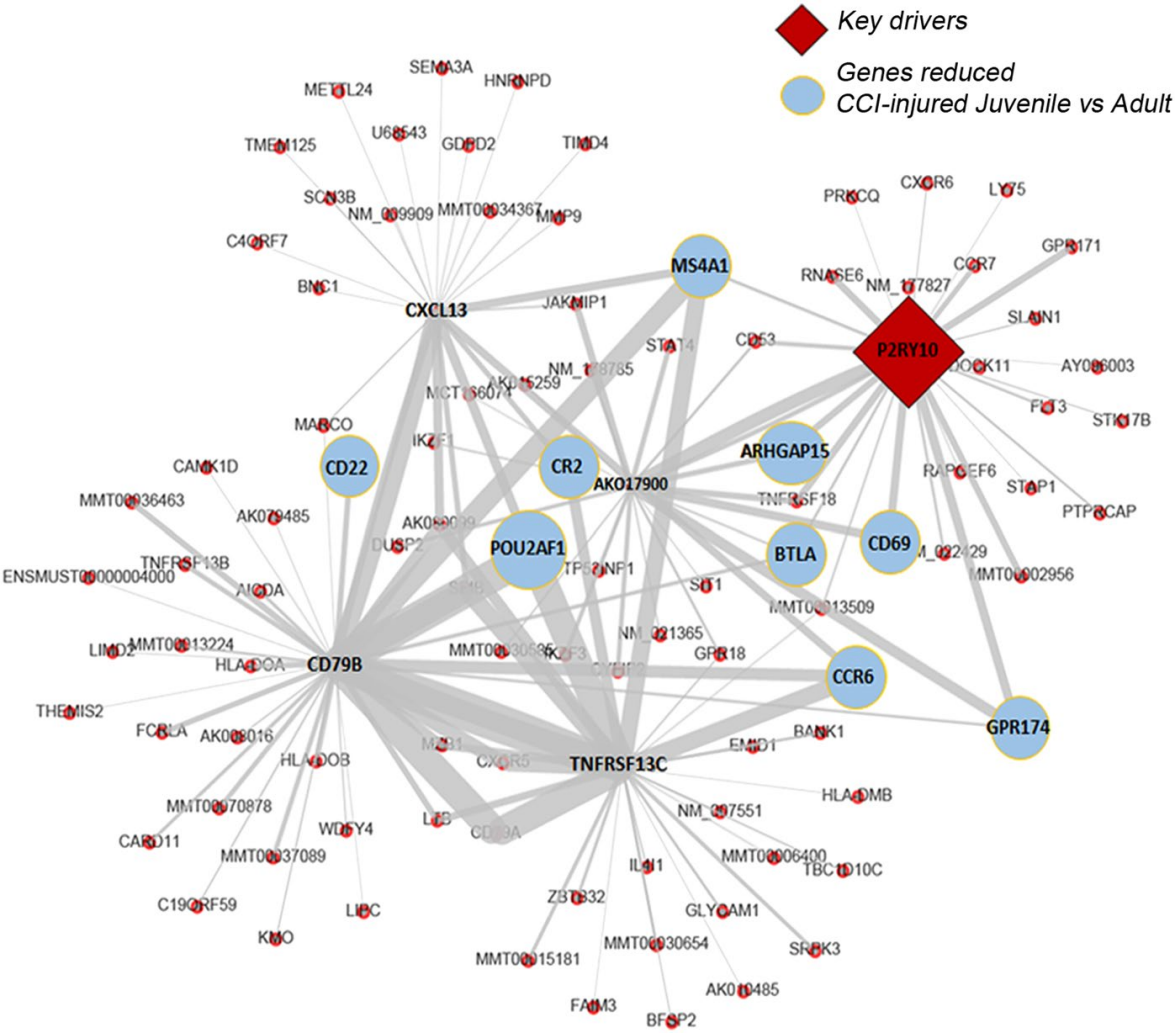
P2ry10 is a key driver of the juvenile gene profile following CCI injury. Mergeomics analysis of differentially reduced genes in juvenile vs adult CCI-injured mouse peripheral blood identified significant interactions between genes and identified P2ry10 as a key driver implicated in the genes found to be reduced in the juvenile peripheral immune response to injury. Red diamond, key driver; Blue circles, reduced juvenile genes; red dots, other known neighboring genes within the network.

Weighted Key Driver Analysis (wKDA) using Mergeomics^20^ was used to evaluate potential networks and key regulators of the CCI-injured genes found to be increased and decreased in the juvenile whole blood compared to adult. wKDA identified a network within the 130 decreased genes to be comprised of *Cd22*, complement C3d receptor 2 (*Cr2)*, POU domain class 2-associating factor 1 *(Pou2af1)*, B-lymphocyte antigen CD20 (*Ms4a1)*, Rho GTPase Activating Protein 15 *(Arhgap15)*, B and T lymphocyte associated (*Btla), Cd69, Ccr6*, G protein-coupled receptor 174 (*Gpr174)* and P2Y receptor 10 *(P2ry10)*. *P2ry10*, a GPCR high affinity receptor for the bioactive lipid lysophosphatidylserine- LysoPS^21^ and a novel conditional danger receptor^22^, was found to be a key driver of this network (Fig. 3). No network was identified amongst the 60 increased genes.

### Age-dependent gene ontology analysis of sham peripheral immune system

**Figure 4:**
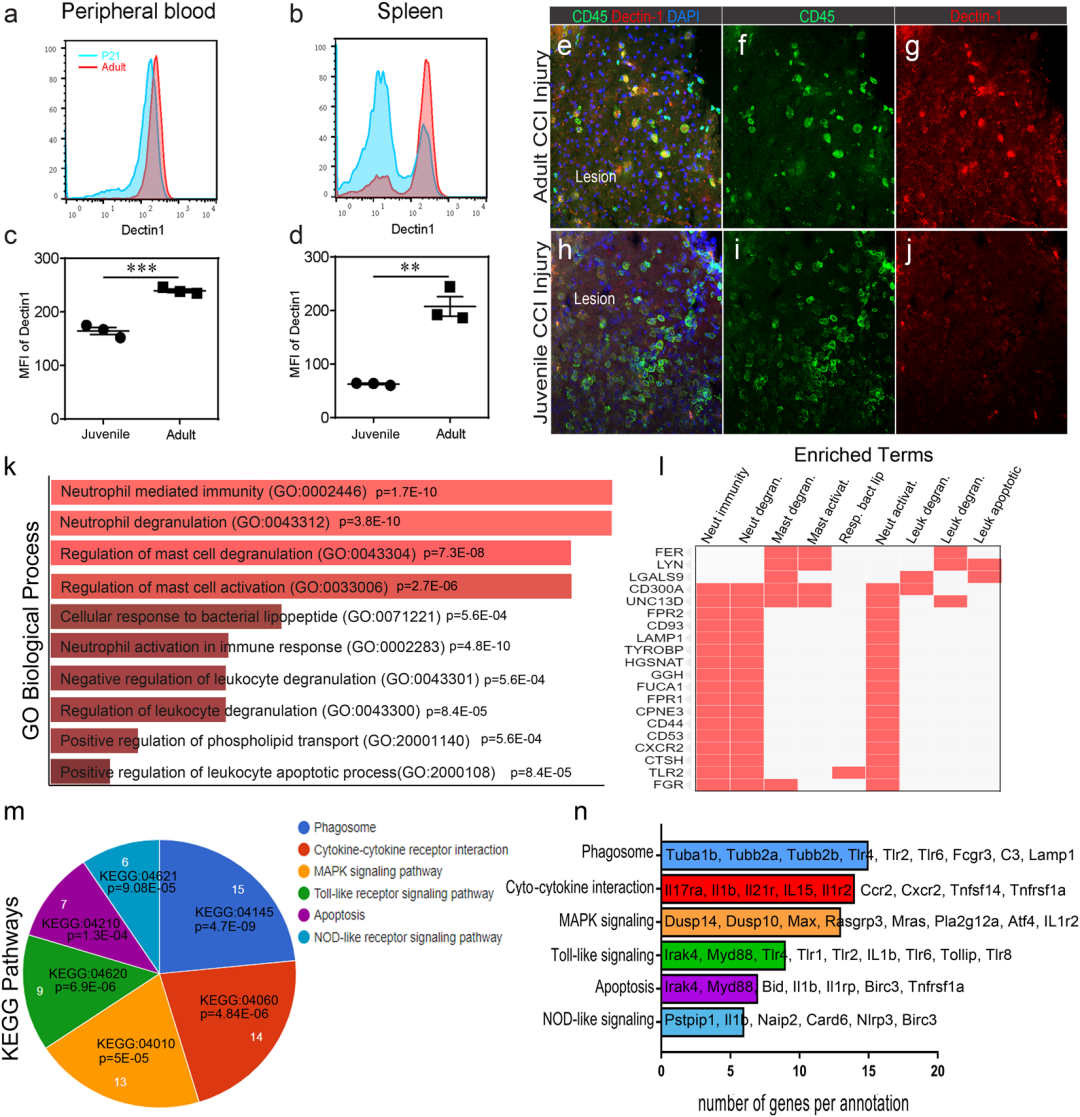
Age-dependent Dectin-1 protein expression and Gene Ontology analysis of sham whole blood. (**a**–**d**) Flow cytometry analysis showed reduced dectin-1 protein expression on Ly6G+ neutrophils in juvenile peripheral blood and spleen compared to adult. (**e**–**g**) Infiltrating CD45+ cells in the lesion of the adult brain also express dectin-1. (**h**–**j**) Dectin-1 expression is decreased in infiltrating CD45+ cells in the lesion of the juvenile brain. (**k**,**l**) Enrichr GO Biological Process analysis of significantly downregulated genes in juvenile vs adult sham peripheral blood. (**m**,**n**) KEGG pathway analysis of significantly downregulated genes in juvenile vs adult sham peripheral blood. P values were calculated using the hypergeometric method.

Lastly, we examined the protein expression of pattern recognition receptor, dectin-1 (*Clec7a*)^23^, which showed reduced RNAseq expression in both sham and 4d CCI-injured juvenile whole blood compared to respective adult mice samples. Dectin-1, a c-type lectin, plays a key role in innate immunity through Toll-like receptor and Nod protein interactions to regulate phagocytosis, ROS and cytokine production^24^. Immune cells were isolated from the spleen and peripheral whole blood of naïve juvenile and adult mice then immuno-stained for Ly6G and dectin-1 protein. Using flow cytometry analysis, we found a significant reduction in dectin-1 expression on Ly6G-positive juvenile neutrophils compared to adult cells from both the peripheral blood (164.7 +/− 11.7 vs 239.7 +/− 5.7 MFI) (Fig. 4a,c) and spleen (62.6 +/− 2.2 vs 207.3 +/− 31.9 MFI) (Fig. 4b,d). Juvenile and adult brain cortices were then processed and immune-stained for CD45 and dectin-1 at 4d post-CCI injury. Infiltrating CD45-positive cells were observed in the adult CCI-injured ipsilateral lesion cavity and showed prominent expression of dectin-1 (Fig. 4e–g), which was absent or reduced on CD45-positive juvenile cells (Fig. 4h–j). Dectin-1 expression was not observed in the un-injured contralateral cortex at either age (data not shown).

Due to these observations, we further analyzed which biological processes were distinct in juvenile sham mice that may impact their initial response to trauma. Using gene ontology we found a number of the 521 genes that were significantly reduced in juvenile sham compared to adult sham whole blood (Fig. 1h,i) to be associated with GO biological processes that included neutrophil and mast cell mediated immunity and degranulation (Fig. 4k,l). Interestingly, a number of these genes were also identified within KEGG pathways detailing phagosome, MAPK, Toll-like and NOD-like receptor signaling (Fig. 4m,n). These differences could have a significant impact on TBI-induced recruitment and inflammatory status, particularly in the neutrophil response. Overall, our findings implicate a potential divergent age-dependent role for pattern/danger recognition receptors and MAPK signaling in the peripheral immune response following TBI.

## Discussion

The findings from this study identify a novel divergent transcriptomic profile in the juvenile immune system that is distinct from the TBI-injured and un-injured adult murine system. Using next-generation sequencing we performed a genome-wide assessment of gene expression and related biological and signaling processes. Most profoundly, we observed a reduction in 521 genes in sham juvenile whole blood compared to sham adults, which were associated with MAPK, Toll-like and NOD-like receptor signaling as well as neutrophil and mast cell function. TLRs and NLRs are pattern-recognition receptors (PRRs) that play a key role in the control of inflammation and are elicited in response to bacteria or damage-associated molecular patterns (DAMPS) which are released by dying cells into circulation after trauma^25,26^. Moreover, we find the protein expression of Dectin-1 is reduced on juvenile neutrophils isolated from the peripheral blood and spleen. Dectin-1 is a c-type lectin receptor (CLR) that senses β-glucans associated with fungal pathogen. However, roles for Dectin-1 in the response to broader damage associated molecular patterns and its role as an “alarmin” have also been described^23,27^. In the CNS, Dectin-1 triggers neuroinflammation that promotes axonal injury and tissue pathology in spinal cord injury^28^. Beyond Dectin-1, the regulation of genes associated with innate immune system and pattern recognition receptor (PRR) signaling suggests that immune system activation may be developmentally regulated and suppression of PRRs in the juvenile circulation may have an immediate impact on tissue damage following brain trauma. Indeed, our recent findings demonstrate reduced injury severity that correlates with improved BBB integrity, functional behavior and cerebral blood flow in juvenile compared to adult mice after CCI injury^15^.

Analysis of the 4 day CCI-injured whole blood gene expression was also performed. Given recent evidence that neutrophil recruitment and retention is prolonged in the juvenile brain which correlates with chronic changes in cortical tissue loss^3^, we anticipated enhanced expression of genes involved in inflammation or neutrophil function. While few genes were found to be up- or down-regulated in the adult injured compared to adult sham whole blood (14 vs 22, respectively), 238 genes were notably increased in the juvenile injured samples. A number of these genes associated with metabolic (GO:0008152), transport (GO:0006810), adhesion (GO:0007155) and cell cycle (GO:0007049) processes but not inflammation (data not shown). With the exception of lysosomal protein transmembrane 4 Beta (*Laptm4b)*, none of these genes were elevated over adult CCI-injured samples suggesting their upregulation was related to the maturation status of the immune system following TBI challenge. Interestingly, *Laptm4b* is a putative oncogene^29^ that could promote cell survival and influence leukocyte life-span both in circulation and in the damaged neural tissue after injury. The most striking GO biological processes we identified were related to the downregulated CCI-injured juvenile genes compared to CCI-injured adult. These genes associated with inflammation, migration, adhesion and aopototic processes. While these differences are most likely due to developmental status, it remains unclear whether the seemingly immature and/or anti-inflammatory state of the juvenile peripheral immune system could paradoxically aid in early neuroprotection at the same time enhance immune cell life span and maturation resulting in late-stage activation.

Key driver analysis also revealed *P2ry10*, a G-protein coupled receptor (GPCR), as an important regulator of the gene network that includes GPR174 and shows reduced expression in the CCI-injured juvenile whole blood. P2RY10 has recently been identified as a novel conditional DAMP receptor that binds lysophospholipids (LPL)^22^. Both P2RY10 and GPR174 are activated by bioactive lipid lysophosphatidylserine (LysoPS), which may modulate T reg function^30,31^. Although, P2RY10 is upregulated on CD4 T cells in an EAE model^32^, its role in the immune system has yet to be determined. Both the GO biological processes and key driver analysis suggest the age-dependent transcriptomic profile of CCI-injured whole blood may reflect differences in gene expression involved in T and B cell receptor signaling. Interestingly, the absence of the adaptive arm of the immune system using *Rag1*−/− mice or a lymphocyte sequester is not protective following TBI^33,34^. However, the age-dependent role of T cell subsets and their influences over neutrophil or monocyte recruitment and function needs further investigation.

Lastly, a number of genes with reduced expression in both juvenile sham vs adult sham and juvenile CCI-injured vs adult CCI-injured cells show association with MAPK signaling, including *Ets, Myc, Il1r2, M-Ras* and *Atf4*. MAPK is activated by the innate immune response via TLRs or NLRs resulting in downstream p38/JNK and inflammatory signaling. MAPK signal transduction is a critical regulator of brain inflammation and is currently considered a major therapeutic target^35^. Overall, our findings demonstrate a distinct, age-dependent transcriptomic profile in the immune system following TBI. A reduction in pattern recognition receptor and MAPK signaling, in particular, may play a significant role in the survival, migration and inflammatory response of the juvenile immune cells which could influence acute and chronic recovery from brain trauma.

## Materials and Methods

### Animals

Mice were housed in an AAALAC accredited, virus/specific antigen-free facility with a 12 h light-dark cycle where food and water were provided ad libitum. CD1 mice were purchased from Charles Rivers and bred to generate the appropriate numbers of mice which were reared until age P21 and P60–P75 as described^15^. All experiments were conducted in compliance with the NIH Guide for the Care and Use of Laboratory Animals and under the approval of the Virginia Tech Institutional Animal Care and Use Committee (IACUC; #15-063) and the Virginia Maryland College of Veterinary Medicine.

### Controlled cortical impact (CCI) injury, whole blood extraction and cell isolation

Animals were prepared for CCI as previously described^36^. Male CD1 mice at postnatal (P) day 21 and P60-75 were anesthetized with ketamine (100 mg/kg) and xylazine (10 mg/kg) intraperitoneal injection and positioned in a stereotaxic frame. Body temperature was monitored with a homeothermic blanket system (Harvard apparatus, Lewes, DE) containing a rectal probe and maintained at 37 °C with an autoregulated heating pad. A 4 mm craniotomy was made using a portable drill over the right parietal-temporal cortex (−2.5 mm A/P and 2.0 mm lateral from bregma). Injury was induced by moderate CCI using the *e*CCI- 6.3 device (Custom Design & Fabrication; 3 mm beveled steel impact tip) at a velocity of 5.0 +/− 0.3 m/s, 1.0 mm depth and 150 ms impact duration^19,37^. Sham controls received craniotomy only.

### Whole blood isolation and analysis

Whole blood was isolated using cardiac puncture under anesthesia and centrifuged at 2,000 × g for 10 minutes at 4 °C to separate serum from cell fraction. The pelleted cell fraction was placed in Trizol (Tri Reagent BD, Sigma-Aldrich) then processed for RNA isolation according to manufacturer’s instructions. RNA quantification was carried out by measuring absorbance with spectrophotometer ND-1000 (NanoDrop). Total RNA from blood cell fraction was pooled from 3 mice and run in technical triplicate. Globin rRNA was depleted using the Globin-Zero Gold rRNA Removal Kit (Illumina). For FACS analysis: Peripheral blood and spleen were harvested from naïve P21 and adult mice, and single cell suspensions were prepared. The cells were then stained with anti-Ly6G and anti-Dectin1 antibodies (BD Biosciences, cat#127607 and cat#144306, respectively) followed by flow cytometry examination. Dectin1 expression within Ly6G^+^ neutrophil population was analyzed^38^. Samples were analyzed with a FACS Canto II (BD Biosciences). FACS plots shown were analyzed with FlowJo (Ashland, OR).

### Next generation RNAseq and analysis

RNA-seq libraries were constructed according to Illumina protocol and sequenced with the Illumina Hiseq. 2000. Sequencing data was analyzed using the public server at usegalaxy.org ^39^. Using TopHat (version 2.0.3), all the 101 bp pair-end reads were mapped to the mouse reference genome (mm9)^40^. Genome annotation files with GTF format for Known Genes were downloaded from UCSC. Fragments per kilobase of transcript per million reads (FPKM) values were calculated for each gene on useGalaxy.org and Cufflinks software (version 2.2.1) with default parameters^41^. Significant changes between groups were calculated using Cuffdiff (version 2.2.1) and normalized using geometric method. The files generated by the Cuffdiff program were then passed to the Cummerbund, an R Core Team package https://www.R-project.org/ version 3.1.2 used to determine the significantly differentially expressed genes (FDR < 0.05) and to visualize the output. Heat maps were generated using R version 3.5.2. The red color indicates high expression and green color indicates low expression. The color code for all figures was generated using log10(FPKM + 1). The FPKM stands for ‘fragments Per Kilobase of transcript per Million mapped reads. GO enrichment analyses of significantly upregulated and downregulated genes were performed using the GeneCodis functional annotation tool^16–18^. Enrichments in GO Biological Process and KEGG Pathways were calculated to identify pathways differentially regulated in juvenile vs adult peripheral blood. Significant pathways were determined using the chi square test (chi < 0.05). For Ingenuity Pathway Analysis (IPA), gene expression data were converted to fold change comparing the adult and juvenile data. All values ± 2-fold change in expression were considered significant for IPA. These parameters yielded 1173 mapped genes for nonbiased analysis. Mergeomics was used to identify any key drivers in the genes that were significantly increased and decreased in CCI-injured juvenile whole blood compared to adult. The wKDA depth was set at 1 and default incoming and outgoing directionality, minimum overlap of 0.33 and edge factor 0.5 were used. Genes were compared against the blood tissue-specific Bayesian network.

### Immunohistochemistry

Freshly dissected whole brain was snap frozen and cryosectioned in serial 30 µm sections. Sections were fixed with 10% buffered formalin, washed 3 times in 1XPBS and block in 2% cold water fish gelatin (Sigma, Inc) in 0.2% triton for one hour. Sections were then exposed to rat anti-dectin-1 and rabbit anti-CD45 (Cell signaling, Inc) antibody (1:100) in block overnight, washed with 1XPBS then treated with anti-rat alexFluor594 and anti-rabbit 488 for one hour. Sections were further washed in 1X PBS then mounted in media with DAPI counter-stain (SouthernBiotech). Images were acquired using a Zeiss 880 confocal microscope (Carl-Zeiss, Oberkochen, Germany).

### Experimental design and statistical analysis

Data was graphed using GraphPad Prism, version 7 XML (GraphPad Software, Inc., San Diego, CA). Student’s unpaired two-tailed - test was used for comparison of two experimental groups. Changes were identified as significant if P was less than 0.05. Mean values were reported together with the standard error of mean (SEM). Sample size was determined based on an effect size measured for each outcome by pilot or prior studies. G*Power 3 (Universitat Dusseldorf, Germany) was used to retrieve sample size using an acceptable power range between 80–90%.

## Supplementary information

supplementary Table 1

## Data Availability

The datasets generated during and/or analyzed during the current study are available from the corresponding author on reasonable request.

## References

[1] Nizamutdinov Damir, Shapiro Lee (2017). Overview of Traumatic Brain Injury: An Immunological Context. Brain Sciences.

[2] McKee CA, Lukens JR (2016). Emerging Roles for the Immune System in Traumatic Brain Injury. Front Immunol.

[3] Claus CP (2010). Age is a determinant of leukocyte infiltration and loss of cortical volume after traumatic brain injury. Dev Neurosci.

[4] Anthony DC, Bolton SJ, Fearn S, Perry VH (1997). Age-related effects of interleukin-1 beta on polymorphonuclear neutrophil-dependent increases in blood-brain barrier permeability in rats. Brain.

[5] Anthony D (1998). CXC chemokines generate age-related increases in neutrophil-mediated brain inflammation and blood-brain barrier breakdown. Curr Biol.

[6] Corps KN, Roth TL, McGavern DB (2015). Inflammation and neuroprotection in traumatic brain injury. JAMA Neurol.

[7] Kaur P, Sharma S (2018). Recent Advances in Pathophysiology of Traumatic Brain Injury. Curr Neuropharmacol.

[8] Gyoneva S, Ransohoff RM (2015). Inflammatory reaction after traumatic brain injury: therapeutic potential of targeting cell-cell communication by chemokines. Trends Pharmacol Sci.

[9] Faden AI, Loane DJ (2015). Chronic neurodegeneration after traumatic brain injury: Alzheimer disease, chronic traumatic encephalopathy, or persistent neuroinflammation?. Neurotherapeutics.

[10] Hinson HE, Rowell S, Schreiber M (2015). Clinical evidence of inflammation driving secondary brain injury: a systematic review. J Trauma Acute Care Surg.

[11] Xiong Y, Mahmood A, Chopp M (2009). Emerging treatments for traumatic brain injury. Expert Opin Emerg Drugs.

[12] Woodcock T, Morganti-Kossmann MC (2013). The role of markers of inflammation in traumatic brain injury. Front Neurol.

[13] Levin HS, Eisenberg HM, Wigg NR, Kobayashi K (1982). Memory and intellectual ability after head injury in children and adolescents. Neurosurgery.

[14] Luerssen TG, Klauber MR, Marshall LF (1988). Outcome from head injury related to patient’s age. A longitudinal prospective study of adult and pediatric head injury. J Neurosurg.

[15] Brickler TR (2018). Angiopoietin/Tie2 Axis Regulates the Age-at-Injury Cerebrovascular Response to Traumatic Brain Injury. J Neurosci.

[16] Tabas-Madrid D, Nogales-Cadenas R, Pascual-Montano A (2012). GeneCodis3: a non-redundant and modular enrichment analysis tool for functional genomics. Nucleic Acids Res.

[17] Nogales-Cadenas R (2009). GeneCodis: interpreting gene lists through enrichment analysis and integration of diverse biological information. Nucleic Acids Res.

[18] Carmona-Saez P, Chagoyen M, Tirado F, Carazo JM, Pascual-Montano A (2007). GENECODIS: a web-based tool for finding significant concurrent annotations in gene lists. Genome Biol.

[19] Theus MH, Ricard J, Bethea JR, Liebl DJ (2010). EphB3 limits the expansion of neural progenitor cells in the subventricular zone by regulating p53 during homeostasis and following traumatic brain injury. Stem Cells.

[20] Arneson D, Bhattacharya A, Shu L, Makinen VP, Yang X (2016). Mergeomics: a web server for identifying pathological pathways, networks, and key regulators via multidimensional data integration. BMC Genomics.

[21] Inoue A (2012). TGFalpha shedding assay: an accurate and versatile method for detecting GPCR activation. Nat Methods.

[22] Wang X (2016). Lysophospholipid Receptors, as Novel Conditional Danger Receptors and Homeostatic Receptors Modulate Inflammation-Novel Paradigm and Therapeutic Potential. J Cardiovasc Transl Res.

[23] Gensel JC (2015). Toll-Like Receptors and Dectin-1, a C-Type Lectin Receptor, Trigger Divergent Functions in CNS Macrophages. J Neurosci.

[24] Underhill DM (2007). Collaboration between the innate immune receptors dectin-1, TLRs, and Nods. Immunol Rev.

[25] Kigerl KA, de Rivero Vaccari JP, Dietrich WD, Popovich PG, Keane RW (2014). Pattern recognition receptors and central nervous system repair. Exp Neurol.

[26] Gadani SP, Walsh JT, Lukens JR, Kipnis J (2015). Dealing with Danger in the CNS: The Response of the Immune System to Injury. Neuron.

[27] Baldwin KT, Carbajal KS, Segal BM, Giger RJ (2015). Neuroinflammation triggered by beta-glucan/dectin-1 signaling enables CNS axon regeneration. Proc Natl Acad Sci USA.

[28] Vijaya Kumar DK, Eimer WA, Ramakrishnan S (2015). Specificity of Toll-Like Receptor 2 and Dectin-1 Signaling in CNS Macrophages. J Neurosci.

[29] Meng Y (2016). LAPTM4B: an oncogene in various solid tumors and its functions. Oncogene.

[30] Shao Ying, Nanayakkara Gayani, Cheng Jiali, Cueto Ramon, Yang William Y., Park Joon-Young, Wang Hong, Yang Xiaofeng (2018). Lysophospholipids and Their Receptors Serve as Conditional DAMPs and DAMP Receptors in Tissue Oxidative and Inflammatory Injury. Antioxidants & Redox Signaling.

[31] Stark Regina, Wesselink Thomas H., Behr Felix M., Kragten Natasja A. M., Arens Ramon, Koch-Nolte Friedrich, van Gisbergen Klaas P. J. M., van Lier René A. W. (2018). TRMmaintenance is regulated by tissue damage via P2RX7. Science Immunology.

[32] Kaur H (2017). Single-cell profiling reveals heterogeneity and functional patterning of GPCR expression in the vascular system. Nat Commun.

[33] Weckbach S (2012). Challenging the role of adaptive immunity in neurotrauma: Rag1(−/−) mice lacking mature B and T cells do not show neuroprotection after closed head injury. J Neurotrauma.

[34] Mencl S (2014). FTY720 does not protect from traumatic brain injury in mice despite reducing posttraumatic inflammation. J Neuroimmunol.

[35] Kaminska B, Gozdz A, Zawadzka M, Ellert-Miklaszewska A, Lipko M (2009). MAPK signal transduction underlying brain inflammation and gliosis as therapeutic target. Anat Rec (Hoboken).

[36] Brickler T (2016). Nonessential Role for the NLRP1 Inflammasome Complex in a Murine Model of Traumatic Brain Injury. Mediators Inflamm.

[37] Baumann G, Travieso L, Liebl DJ, Theus MH (2013). Pronounced hypoxia in the subventricular zone following traumatic brain injury and the neural stem/progenitor cell response. Experimental biology and medicine.

[38] Zhang Y, Geng S, Prasad GL, Li L (2018). Suppression of Neutrophil Antimicrobial Functions by Total Particulate Matter From Cigarette Smoke. Front Immunol.

[39] Afgan E (2018). The Galaxy platform for accessible, reproducible and collaborative biomedical analyses: 2018 update. Nucleic Acids Res.

[40] Trapnell C, Pachter L, Salzberg SL (2009). TopHat: discovering splice junctions with RNA-Seq. Bioinformatics.

[41] Trapnell C (2010). Transcript assembly and quantification by RNA-Seq reveals unannotated transcripts and isoform switching during cell differentiation. Nat Biotechnol.

[42] Kramer A, Green J, Pollard J, Tugendreich S (2014). Causal analysis approaches in Ingenuity Pathway Analysis. Bioinformatics.

