# Supplementary material for: Divergent age-dependent peripheral immune transcriptomic profile following traumatic brain injury
Source: Sci Rep. 2019 Jun 12;9:8564. doi: 10.1038/s41598-019-45089-z (PMC6561964; doi:10.1038/s41598-019-45089-z)

**Divergent age-dependent peripheral immune transcriptomic profile following traumatic brain injury**

Amanda Hazy^1^, Lauren Bochicchio^1^, Andrea Oliver^1^, Eric Xie^2^, Shuo Geng^6^, Thomas Brickler^1^, Hehuang Xie^1,3^, Liwu Li^6^, Irving C. Allen^1^ and Michelle H Theus^1,4,5^

^1^The Department of Biomedical Sciences and Pathobiology, Virginia Tech, 970 Washington Street, Blacksburg, VA, 24061 USA

^2^The Department of Statistics, Virginia Tech, 250 Drillfield Drive, Blacksburg, VA, 24061 USA

^3^Biocomplexity Institute, Virginia Tech, 1015 Life Science Circle, Blacksburg, VA, 24061 USA

^4^School of Neuroscience, Virginia Tech, Blacksburg VA 24061, USA

^5^Center for Regenerative Medicine, College of Veterinary Medicine, Blacksburg, Virginia, 24061, USA

^6^The Department of Biological Sciences, College of Science, Virginia Tech, 970 Washington Street, Blacksburg, VA, 24061 USA

Supplementary Table 1

**Corresponding author:**

Michelle H. Theus, Ph.D.

Associate Professor

Department of Biomedical Sciences and Pathobiology

College of Veterinary Medicine, Virginia Tech

970 Washington Street SW (MC0910)

Blacksburg, VA 24061

Tel. 540-231-0909; Fax 540-231-7425; E-mail: [**mtheus@vt.edu**](mailto:mtheus@vt.edu)

**Supplementary Table 1.** Gene list of common up- and down-regulated genes amongst both sham and CCI-injured samples. FDR<0.05 was used to identify those that were significant.


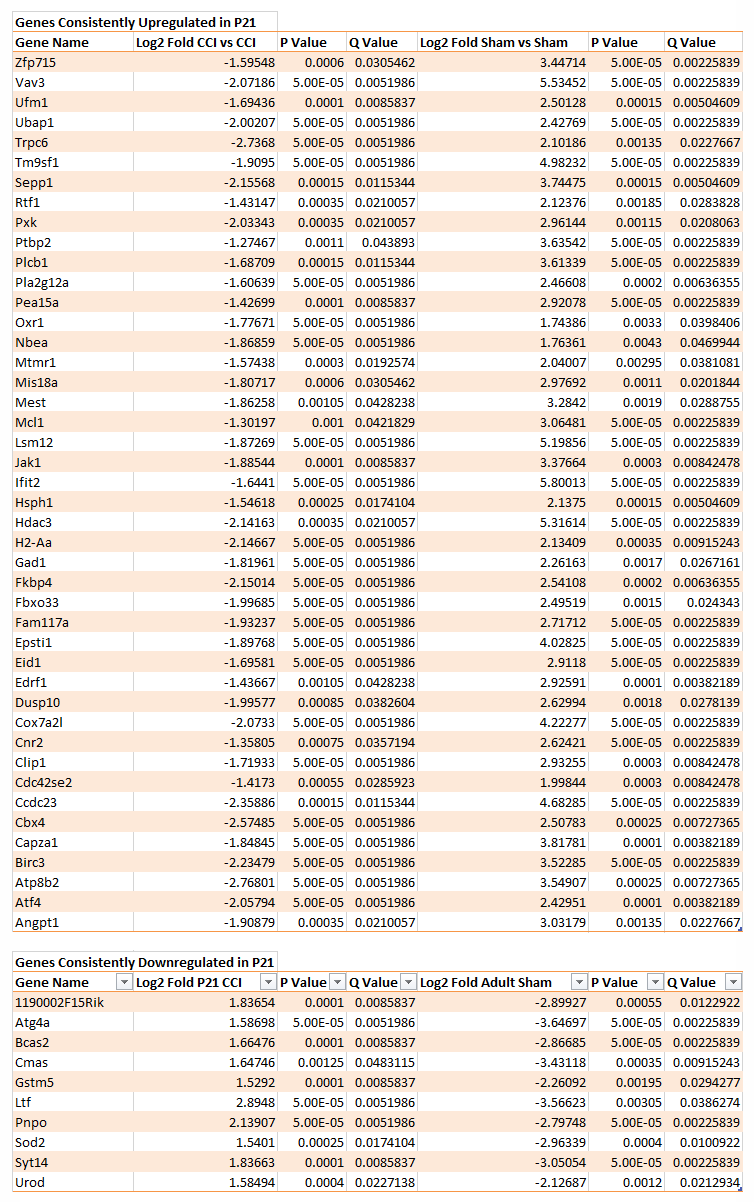

Supplement: Supplementary file 1 — supplementary Table 1 [file 41598_2019_45089_MOESM1_ESM.docx]
